# Supplementary material for: AffyMAPSDetector: a software tool to characterize Affymetrix GeneChip™ expression arrays with respect to SNPs
Source: BMC Bioinformatics. 2007 Jul 30;8:276. doi: 10.1186/1471-2105-8-276 (PMC1959249; doi:10.1186/1471-2105-8-276)
Supplement: Additional file 13 — AffyMAPSDetector v1 source code. AffyMAPSDetector source code. [file 1471-2105-8-276-S13.zip › AffyMAPSDetector_v1_src/doc/index-files/index-5.html]

E-Index


|  |  |  |  |
| --- | --- | --- | --- |
| **Overview** \| Package \| Class \| Use \| **Tree** \| **Deprecated** \| **Index** \| **Help** | | |  |
| **PREV LETTER**   **NEXT LETTER** | **FRAMES**    **NO FRAMES**     **All Classes** |  |  |


A B C D E F G H I J L M N O P R S T V W X Y 

---


## **E**

**E** - Static variable in class affychip.snpdetector.util.PmMmHeatMapViewer: **ErrorMessageBox** - class jwf.ErrorMessageBox.: Displays a list of error messages and blocks until ok is pressed. **ErrorMessageBox()** - Constructor for class jwf.ErrorMessageBox: Construct a dialog with no parent. **ErrorMessageBox(Frame)** - Constructor for class jwf.ErrorMessageBox: Construct a dialog with a frame parent. **ErrorMessageBox(Dialog)** - Constructor for class jwf.ErrorMessageBox: Construct a dialog with a dialog parent. **eFetch(String, GeneInfo)** - Method in class affychip.snpdetector.model.OptimizedSnpDetector: This method accesses a gene with a given accession number (from GenBank) and locus-link-id online and searches NCBI snp database for existence of SNPs in this gene's nucleotide-sequence. **eFetchRsInfoUrl** - Static variable in class affychip.snpdetector.model.OptimizedSnpDetector: **eFetchUrl** - Static variable in class affychip.snpdetector.model.OptimizedSnpDetector: **eLink(String)** - Method in class affychip.snpdetector.model.OptimizedSnpDetector: **eLinkUrl** - Static variable in class affychip.snpdetector.model.OptimizedSnpDetector: **eSearch(String)** - Method in class affychip.snpdetector.model.OptimizedSnpDetector: **eSearchUrl** - Static variable in class affychip.snpdetector.model.OptimizedSnpDetector: **eUtilBase** - Static variable in class affychip.snpdetector.model.OptimizedSnpDetector

---


|  |  |  |  |
| --- | --- | --- | --- |
| **Overview** \| Package \| Class \| Use \| **Tree** \| **Deprecated** \| **Index** \| **Help** | | |  |
| **PREV LETTER**   **NEXT LETTER** | **FRAMES**    **NO FRAMES**     **All Classes** |  |  |


A B C D E F G H I J L M N O P R S T V W X Y 

---
